# Supplementary material for: Food Waste-Assisted Metal Extraction from Printed Circuit Boards: The Aspergillus niger Route
Source: Microorganisms. 2021 Apr 22;9(5):895. doi: 10.3390/microorganisms9050895 (PMC8143491; doi:10.3390/microorganisms9050895)
Supplement: Supplementary file 1 [file microorganisms-09-00895-s001.zip › microorganisms-1189795-supplementary.pdf]

Metals extraction from printed circuit boards using the citric acid produced by Aspergillus niger NRRL 334

Carlotta Alias[1]°, Daniela Bulgari[2]°*, Fabjola Bilo[3], Laura Borgese[3], Alessandra Gianoncelli[4], Giovanni Ribaudo[4], Emanuela Gobbi[2], Ivano Alessandri[5,6,7]

Corresponding author: Daniela Bulgari

[2] Agri-food and Environmental Microbiology Platform (PiMiAA), Department of Molecular and Translational Medicine, University of Brescia, Brescia 25123, Italy

Address: viale Europa, 11, 25123, Brescia, Italy

E-mail: daniela.bulgari@unibs.it

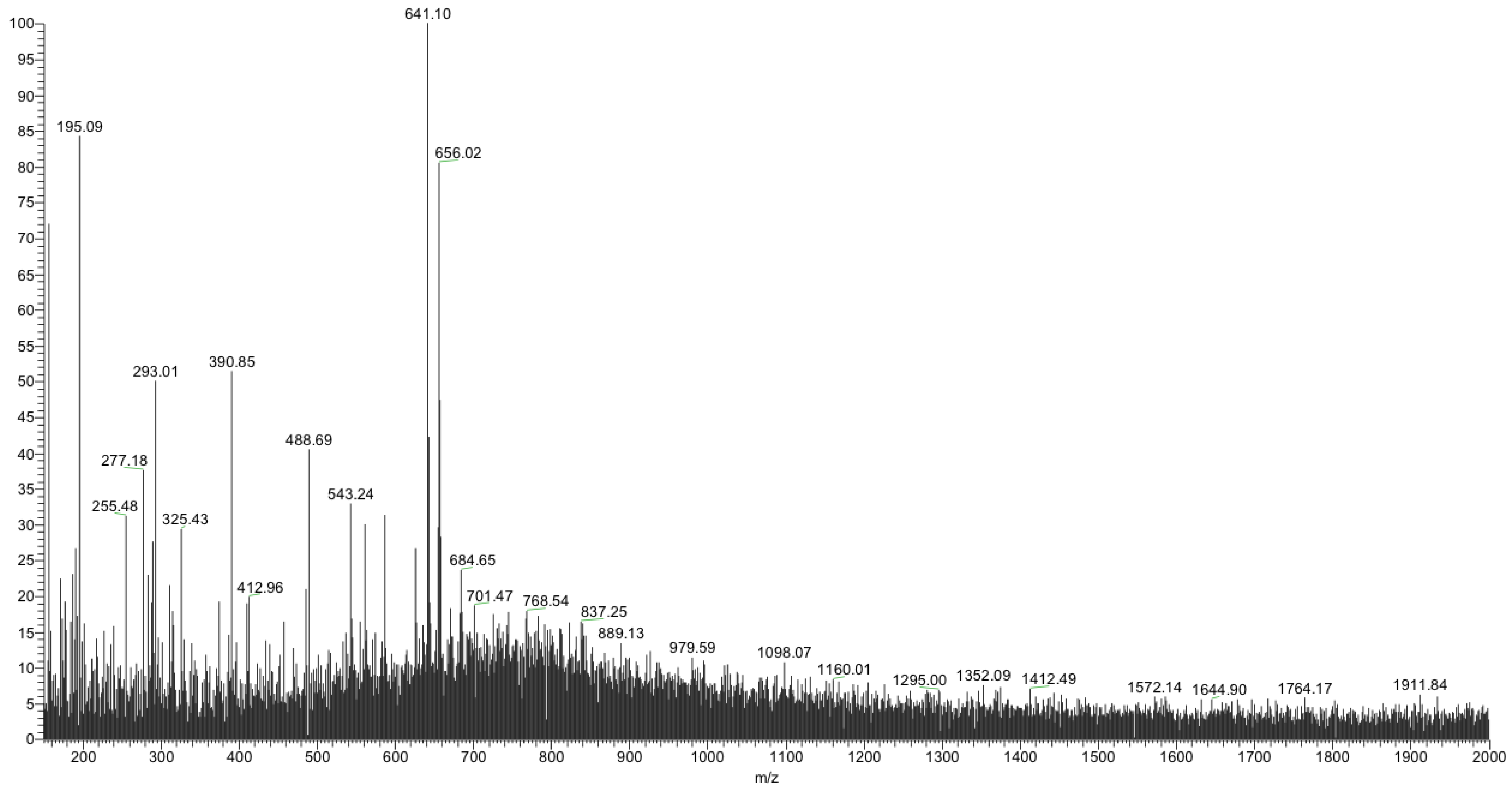

Online Resource 1. Negative ionization ESI-MS analysis of control solution (1:1000 in methanol).
